# Photoelectrochemical water splitting strongly enhanced in fast-grown ZnO nanotree and nanocluster structures†
†Electronic supplementary information (ESI) available. See DOI: 10.1039/c6ta02788a



**DOI:** 10.1039/c6ta02788a

**Published:** 2016-06-01

**Authors:** Xin Ren, Abhijeet Sangle, Siyuan Zhang, Shuai Yuan, Yin Zhao, Liyi Shi, Robert L. Z. Hoye, Seungho Cho, Dongdong Li, Judith L. MacManus-Driscoll

**Affiliations:** a Research Center for Nanoscience and Technology , Shanghai University , 99 Shangda Road , Shanghai 200444 , China . Email: renxin108@shu.edu.cn ; Fax: +86 21 66137197 ; Tel: +86 21 66137197; b Department of Materials Science and Metallurgy , University of Cambridge , 27 Charles Babbage Road , Cambridge CB3 0FS , UK . Email: jld35@cam.ac.uk ; Fax: +44 (0)1223334468 ; Tel: +44 (0)1223334468; c Shanghai Advanced Research Institute , Chinese Academy of Sciences , 99 Haike Road, Zhangjiang Hi-Tech Park , Shanghai 201210 , China

## Abstract

We demonstrate selective growth of ZnO branched nanostructures: from nanorod clusters (with branches parallel to parent rods) to nanotrees (with branches perpendicular to parent rods). The growth of these structures was realized using a three-step approach: electrodeposition of nanorods (NRs), followed by the sputtering of ZnO seed layers, followed by the growth of branched arms using hydrothermal growth. The density, size and direction of the branches were tailored by tuning the deposition parameters. To our knowledge, this is the first report of control of branch direction. The photoelectrochemical (PEC) performance of the ZnO nanostructures follows the order: nanotrees (NTs) > nanorod clusters (NCs) > parent NRs. The NT structure with the best PEC performance also possesses the shortest fabrication period which had never been reported before. The photocurrent of the NT and NC photoelectrodes is 0.67 and 0.56 mA cm^–2^ at 1 V *vs.* Ag/AgCl, respectively, an enhancement of 139% and 100% when compared to the ZnO NR structures. The key reason for the improved performance is shown to be the very large surface-to-volume ratios in the branched nanostructures, which gives rise to enhanced light absorption, improved charge transfer across the nanostructure/electrolyte interfaces to the electrolyte and efficient charge transport within the material.

## Abstract 2

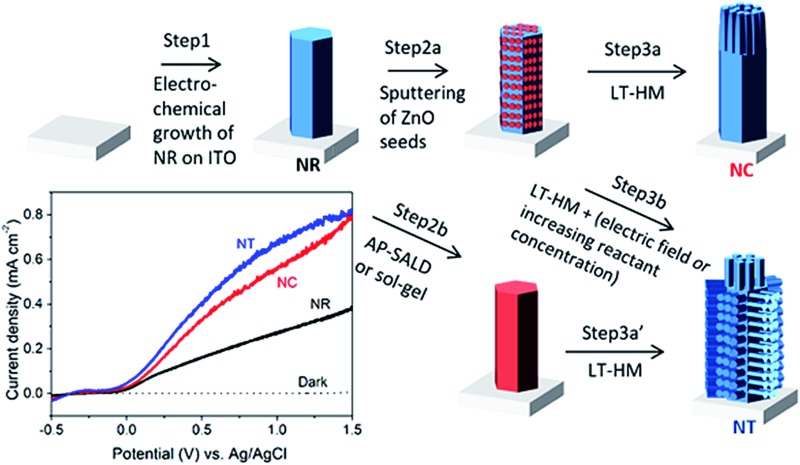
We demonstrated a versatile route to fast-fabricate hierarchical ZnO nanostructures which give rise to high photocurrents.

## Introduction

ZnO, a II–VI semiconductor with a large direct band gap (3.37 eV) and a large exciton binding energy (60 meV), is of considerable interest for various applications, such as piezoelectric transducers,^1,2^ chemical sensors,^3,4^ catalysis,^5,6^ photovoltaics^7–11^ and photoelectrochemical (PEC) water splitting,^12–17^ as a low-cost, earth-abundant and non-toxic material. ZnO is known to be easy to nanostructure^18^ and exhibits a range of quasi-one dimensional (1D) morphologies, such as rods,^19–21^ wires,^22^ tubes^23^ and belts,^24^ which have been prepared by various approaches, including evaporation and condensation processes,^25^ metal–organic chemical vapor deposition,^26^ hydrothermal growth,^27–30^ molecular beam epitaxy^31^ and electrodeposition.^32–35^

In the field of PEC water splitting, much work has focused on 1D nanostructured ZnO photoelectrodes because of enhancements in charge separation, charge transport, and light absorption.^36^ However, the surface area of 1D nanostructures is relatively small, negatively impacting the charge transfer process.^37^ ZnO hierarchical nanostructures, especially branched nanorods are expected to exhibit a more marvelous performance, because they not only have the merits of the 1D nanostructures, but also have larger surface areas for efficient charge transfer.^37^ As a facile, cost-effective and scalable fabrication technique, hydrothermal growth is one of the most common methods to fabricate ZnO hierarchical nanostructures. Xing Sun *et al.* adopted a four-step hydrothermal growth method (seed solution drop-cast + nanowire hydrothermal growth + seed solution drop-cast + nanobranch hydrothermal growth) to synthesize ZnO nanoforests, with a total synthesis period from 14 to 17 h.^38^ Seung Hwan Ko *et al.* applied similar approaches to produce the nanoforest of hierarchical ZnO nanowires, with a total synthesis period from 6 to 20 h.^28^ The architecture of the ZnO hierarchical nanostructures can be tailored by tuning the synthesis parameters. Despite the numerous merits mentioned above, the slow hydrothermal growth process is one of the major obstacles for the commercial application of this technique.

Herein, we demonstrate a versatile route to grow ZnO hierarchical nanostructures. Nanorod clusters (with branches parallel to parent rods) and nanotrees (with branches perpendicular to parent rods) can be selectively grown on indium tin oxide (ITO) coated glass substrates by switching the applied electric field or by changing the solution concentration. Moreover, the period of the fabrication of nanotrees was less than 1 hour under the action of electric field, which is exceedingly shorter than the period of several hours that was commonly reported before.^28,38,39^ Despite the short fabrication period, after short annealing at 450 °C the NT structure exhibited superior PEC water splitting performance. The PEC behavior of both the NC and NT arrays showed enhanced PEC water splitting with photocurrents of 0.56 and 0.67 mA cm^–2^, respectively at an applied potential of 1 V *vs.* Ag/AgCl (3 M KCl). This is higher than that for the reference NR arrays of this work (0.28 mA cm^–2^), and is also superior to those of pristine ZnO NR structures prepared by magnetron sputtering, hydrothermal reaction and electrodeposition (∼0.05–0.3 mA cm^–2^) reported previously.^38–41^ The superior performance observed here for the NC and NT arrays is attributed to the large surface-to-volume ratios of these branched nanostructures.

## Experimental

### Preparation of the different ZnO branched nanostructures

**Fig. 1:**
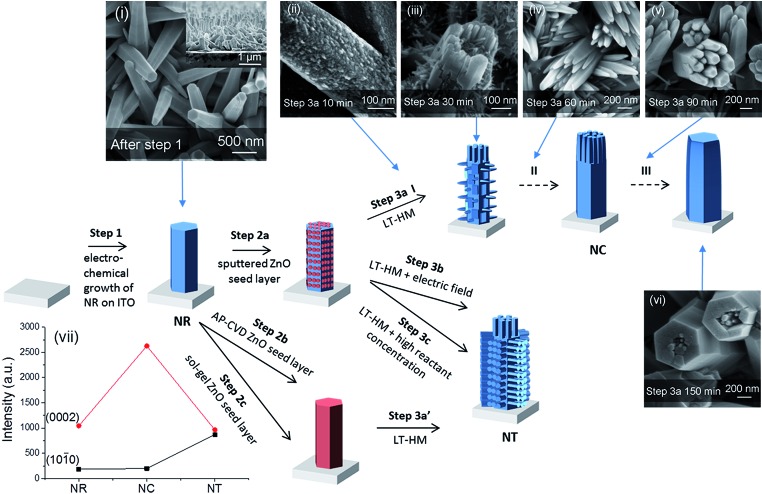
Illustration of different growth processes of nanorod clusters (NCs) and nanotrees (NTs) from nanorods (NRs). Step 1 indicates electrodeposition of NRs. Steps 2a, 2b and 2c indicate deposition of ZnO seed layers by sputtering, by AP-CVD and by sol–gel, respectively. Steps 3a, 3b and 3c indicate growth of branched arms by the low-temperature hydrothermal method (LT-HM) without an electric field (3a and 3a′), and with an electric field (3b), and with no field but a higher concentration LT-HM solution (3c), respectively. I, II, and III indicate three stages, early, medium and final stage, in step 3a. Top view SEM images of (i) as-electrodeposited ZnO NRs (with the cross-section image shown in the inset), (ii–vi) evolution of sputtered seeded ZnO NRs (after step 2a) during the LT-HM process for (ii) 10, (iii) 30, (iv) 60, (v) 90 and (vi) 150 min. (vii) XRD peak intensities of ZnO (1010) (planes parallel to ‘c’) and (0002) (planes perpendicular to ‘c’) of NR (i), NC (iv) and NT (after step 3b) arrays. The XRD patterns are shown in Fig. S1.†

Fig. 1 presents the design and preparation process of the ZnO branched nanostructures. First, the ITO/glass substrates (Praezisions Glas & Optik, 10–15 Ω □^–1^) were cleaned in an ultrasonic bath with acetone and then isopropanol for 30 min for two rounds prior to electrodeposition of ZnO. ZnO NRs were electrodeposited potentiostatically using a Keithley 2400 sourcemeter under a constant voltage of 2 V in 0.01 M zinc nitrate aqueous solution at 85 °C for 40 min (step 1).^42^ An ITO substrate was connected to the cathode, and a platinum foil was employed as the anode. After electrodeposition, the samples were rinsed with deionized water and dried with compressed air.

Next, an Emitech K575X sputter coater was employed to deposit thin (∼5 nm) ZnO films as seed layers onto the samples with 100 mA current for 60 s (step 2a). For comparison, two alternative ways to deposit the seed layer were undertaken, one being atmospheric pressure chemical vapor deposition (AP-CVD) (step 2b)^43^ and the other being the sol–gel method (step 2c) with details described in Fig. S2 and S3.† We schematically show the fabrication process in Fig. 1, with the red color denoting the seed layer. The seed layer has incomplete coverage for the sputtered layer (owing to vapor shadowing effects), but it is complete for the AP-CVD and sol–gel seed layers. As we show in Fig. 1 and discuss in more detail later, the incomplete coverage of the sputtered seed layer results in a lower density of side-wall arms and ultimately elimination of side wall arms, and the NC structure. On the other hand, the NT structure forms and remains stable for the AP-CVD and sol–gel seed layers.

Following coating of the seed layers by the different methods, low temperature hydrothermal growth (LT-HM) of ZnO branches onto the seeded ZnO NRs was undertaken in step 3. To verify the role of the seed layers in the formation of the nanostructures, LT-HM was also undertaken onto the bare ZnO NRs without the seed layer as a comparison. Here, the samples were fixed on a supporting glass slide facing down and immersed in the aqueous solution. In step 3a, 25 mM zinc nitrate and an equivalent amount of hexamethylenetetramine (HMT) at 85 °C were used. Gentle agitation of the solution was employed during hydrothermal growth for times varying from 10 to 150 min. In step 3b a constant voltage of 2.1 V was applied during hydrothermal growth, whereas in step 3c no field was applied, but instead a higher concentration of solution (100 mM zinc nitrate in an equivalent amount of HMT aqueous solution) was used.

After low-temperature hydrothermal synthesis, samples from step 3 were rinsed and kept in a dark and dry air atmosphere. Some of the samples were placed in a furnace and annealed at 450 °C in air for 2 h.

### Materials characterization

The morphology of the nanostructures was observed using a LEO GEMINI 1530VP FEG scanning electron microscope (SEM). The crystalline structure of the deposited materials was measured using a Bruker D8 θ/θ X-ray diffraction (XRD) system with Cu Kα radiation (*λ* = 1.5418 Å). Photoluminescence (PL) measurements were performed at room temperature with an ACCENT RPM 2000 compound semiconductor PL system excited by a laser with a wavelength of 266 nm. UV-visible spectra were recorded in a SHIMADZU UV-2600 UV-VIS spectrophotometer. All the spectra were background subtracted.

### Electrochemical measurements

Mott–Schottky plots were measured in a three-electrode cell using a Pt wire as the counter electrode and standard Ag/AgCl (3 M KCl) as the reference electrode with the use of a PARSTAT 2273 Potentiostat/Galvanostat. A 0.1 M LiClO_4_ carbonate propylene electrolyte was used to prevent ZnO decomposition after deposition.^44^ Each measurement was performed by applying a 20 mV AC sinusoidal signal at a constant applied bias, with a frequency of 1 kHz. The PEC water splitting performance and electrochemical impedance spectra (EIS) of the ZnO nanostructured photoelectrodes were evaluated using an AUTOLAB PGSTAT302N/FRA2 in a three-electrode system with an Ag/AgCl (3 M KCl) reference electrode and a platinum wire counter electrode. The working electrode with an illuminated area of 0.8 cm × 0.8 cm was immersed in 0.5 M Na_2_SO_4_ aqueous solution and illuminated using a 300 W xenon lamp with a light intensity of 100 mW cm^–2^ coupled with an AM 1.5G filter (PLS-SXE300/300UV). Linear sweep voltammetry (LSV) was performed with a voltage scan speed of 0.01 V s^–1^ ranging from –0.5 to 1.5 V. Photocurrent density–time (*J*–*t*) curves were measured at an applied bias of 1.0 V *vs.* the Ag/AgCl electrode. EIS were measured by applying a bias of 0.5 V *vs.* the Ag/AgCl electrode over the frequency range of 10^–1^ to 10^5^ Hz with a 5 mV amplitude. Incident photon-to-current conversion efficiencies (IPCEs) were measured as a function of wavelength from 300 to 450 nm with a three-electrode configuration at 0 V *vs.* Ag/AgCl using an AUTOLAB electrochemical station with the assistance of a QEX10 commercial spectral response system. Each wavelength was held for 3 min before recording the photocurrent.

## Results and discussion

We first compare the different ZnO nanostructures which were grown under the different deposition steps (*i.e.* ZnO NRs (parent rods) + seed layer + growth of ZnO branches by LT-HM, with or without an electric field). We then compare the overall orientations of the resulting branched structures. Next, we analyze the structural formations in relation to the nature of the seed layers applied to parent NR structures, to whether an electric field is applied or not, and to hydrothermal bath solution concentration. Finally, we determine and analyze the water splitting performances of the different nanostructures.

As shown in Fig. 1i, the as-electrodeposited ZnO NRs have an average diameter of 180 nm and average length of 1.5 μm (as shown in the inset), with a packing density of 3 × 10^8^ cm^–2^. The NRs with seeded ZnO layers cannot be distinguished from the as-electrodeposited NRs using SEM.

Fig. 1ii–vi show the evolution process of the ZnO NRs during the LT-HM process (step 3a). After 10 min of incubation, the primary ZnO crystal is already covered by the overgrown ZnO grains, with several nascent branches beginning to grow (Fig. 1ii). After 30 min (Fig. 1iii), the branches grow further, with the branches on the heads of the ZnO parent rods (*i.e.* the (0002) planes) being much larger than those perpendicular to the ZnO parent rods (*i.e.* on the {1010} side facets of the rods). After 60 min (Fig. 1iv), only the branches on the heads of the ZnO parent rods have grown, while those perpendicular to the ZnO parent rods have disappeared. After 90 min (Fig. 1v), the overgrown rods on the heads of the parent ZnO rods are clustered and highly faceted similar to those shown in Fig. 1iv. The {1010} faces (*i.e.* the surfaces) of the rods are very smooth and no rods were observed perpendicular to the parent rods. After 150 min (Fig. 1vi), the overgrown ZnO rods have coalesced and almost completely covered the parent rods.

**Fig. 2:**
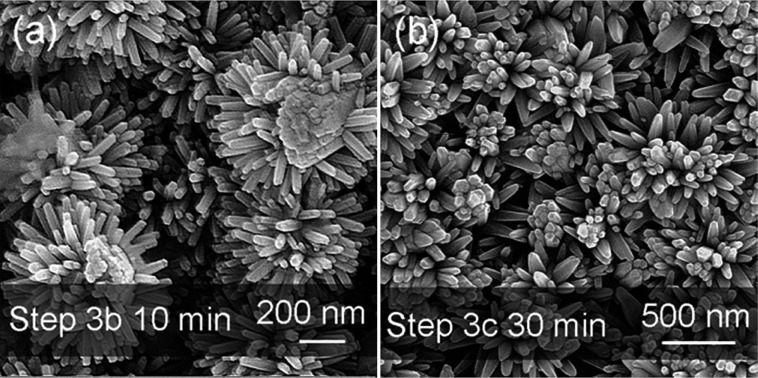
Top view SEM images of the ZnO NT structures produced in step 3b (a) using 25 M zinc nitrate + 25 M HMT LT-HM solution with a constant applied voltage of 2.1 V, and in step 3c (b) using a high concentration LT-HM solution (100 M zinc nitrate + 100 M HMT) *without* an applied electric field.

As Fig. 1 illustrates, the NT structures are formed after step 3b, 3c or 3a′ (corresponding SEM images shown in Fig. 2a, b, S2 and S3†, respectively). Briefly, all the NT structures show the side branches with axes perpendicular to the parent NRs, as well as head branches with axes parallel to the parent NRs. The details of each NT morphology and the differences between them are discussed later.

The different orientations of the branches formed in the NC and NT structures are compared using X-ray analysis (Fig. 1vii). It is observed that some of the NRs preferentially grow in the *c*-axis direction ((0002) planes) compared to perpendicular to ‘c’ ((1010) planes). On the other hand, the NC has a very strong *c*-axis preferred orientation, whereas the side-branched NT has a reduced *c*-axis preferred orientation compared to NRs and NCs. This is consistent with the large density of side-branches which grow perpendicular to the *c*-axis oriented parent rods in the NT structures (Fig. 2).

For the AP-CVD and sol–gel seeded surfaces, after LT-HM growth, NT structures (with similar morphology to Fig. 2, shown in Fig. S2 and S3†) were obtained instead of NC structures (Fig. 1iv and v). While the NT structure has been reported previously,^28,35,45^ as far as we know the NC structure has not. To confirm whether the ZnO seed layer on the parent ZnO NRs played a key role in the formation of the nanostructures, bare ZnO NRs were also subjected to LT-HM with identical parameters to those in step 3a. Fig. S4† shows the nanostructure after 60 min hydrothermal growth. It can be seen that the hydrothermally grown NR is longer and thicker compared with the original parent NR (Fig. 1i). But there is no branch around the NR, verifying the essential role of the ZnO seed layer deposited in step 2 in the formation of branched nanostructures.

In order to understand what leads to the NC *versus* NT structure formation, for the different steps, 3a, 3a′, 3b and 3c, we analyzed the nucleation and growth of side branch structures on the sputter seeded NRs (NCs) *versus* the AP-CVD/sol–gel seeded NRs (NTs), as well as the influence of electric fields/high solution concentration during the LT-HM process (NTs).

It is known that the critical nuclei size r* that determines the growth or dissolution of a deposited crystal can be obtained from the equationr* = 2*Vγ*/3*k*_B_ *T* ln(*S*)where *V* is the molecular volume of the precipitated species, *k*_B_ the Boltzmann constant, *S* the saturation ratio of the reactants, and *γ* the surface free energy per unit surface area. From this equation, we see that the higher the saturation ratio *S*, the smaller the critical nucleus size r*. For a given value of *S*, all sputtered seeds larger than r* will decrease their free energy and hence will form stable nuclei. On the other hand, all seeds smaller than r* will dissolve.

### Growth of NCs on seeded NRs with incomplete coverage (step 3a)

At the beginning of the hydrothermal process, *S* is high, and the critical nuclei size r* is small and so the randomly covered ZnO seed nuclei formed after step 2a are stable all over the parent rod. In step 3a, from the ZnO nuclei the ZnO branches grow both parallel and perpendicular to the parent NR and, at the same time, the parent NR itself also grows. The head branches grow faster than the side branches (step 3ai) since the diffusion route of reactants to the head branches is shorter than to side branches. As the growth of the branches proceeds across step 3a, the reactant OH^–^ ions derived from HMT^46^ and the Zn^2+^ in the solution are gradually consumed by the growth of branches and the parent NR, and also by ZnO particles which precipitate in the solution. *S* decreases due to the depletion of the reactants. Hence, r* increases with growth time, leading to Ostwald ripening. Thus, the larger head branches grow, and the smaller ones (nucleated along the side arms) become unstable and hence dissolve (step 3aii). As r* further increases the overgrowth head branches merge into large ones, with one large rod ultimately forming (step 3aiii).

### Growth of NTs on seeded NRs with complete coverage (step 3a′)

For the AP-CVD/sol–gel seeded NRs (formed after step 2b/2c), the surface of the NRs is completely covered by the ZnO seeds, and so no parent NR is exposed to the solution. Since the parent NR is completely covered by the seeds, the large parent rod cannot influence the growth process of the branched rod. The hierarchical branches grow on the seeded parent NR which acts as the growth substrate. Since the parent NR was completely covered, the side branches, rather than the parent branch, grew.

The morphologies of the NT structures grown from the AP-CVD and sol–gel seeded NRs are shown in Fig. S2 and S3,† respectively. Comparing Fig. S2 with S3,† the branches of the NTs grown from the sol–gel seeded NRs are sparser than those of the NTs grown from the AP-CVD seeded NRs. This is consistent with sparser seeds in the sol–gel case, resulting from the agglomeration of the gel during the sol–gel transition process.

Fig. S3† also shows the relationship between the number of branches and the size of seeds. Comparing Fig. S3a with b,† it can be seen that the seed size in the inset of Fig. S3a† is much smaller, with a small number of seeds reaching up to 8 nm, corresponding to the scarce number of branches shown in Fig. S3a.† This implies that the critical nucleus size r* is around 8 nm for the LT-HM in steps 3a and 3a′.

### Growth of NTs under the action of an electric field (step 3b)

We now discuss the influence of an applied electric field on the growth of the NT structure (step 3b). The conditions are the same as for the growth of the ZnO NRs of Fig. 1ii, except that an electric field was applied during the hydrothermal process. The density of accumulated surface charge on the ZnO seed nuclei (formed by the sputtered seeds in step 3a) is given by*σ* = *Q*/(*r* × *r*) ≈ *V*/*r*where *σ* is the charge density, *Q* is the amount of charge, *V* the electrical potential, and *r* the radius of curvature of the tips of the growing branches. The negative charge on the rods under the application of an electric field during hydrothermal growth attracts Zn^2+^ ions, while OH^–^ ions are supplied both by the HMT and by the H_2_O in the presence of nitrate ions and negative charges:NO_3_^–^ + H_2_O + 2e^–^ → NO_2_^–^ + 2OH^–^

The ZnO needle tips grow faster than the parent NRs due to the higher current density and in turn higher concentration of Zn^2+^ and OH^–^ ions around the tips.^47^ The NT structures finally form due to the faster growth of the needle tips (Fig. 2a). It is observed that the branches are nearly perpendicular to the parent rods. The diameters of the branches are more uniform than those obtained for the NCs (Fig. 1iv) because the charge density at the smaller tips is higher. Hence, the smaller tips grow faster than the larger ones until their sizes become uniform. They are also smaller than for the NC branches and are ∼10 nm compared to 10–50 nm, because the higher concentration of reactants reduces the critical nuclei size r*, increasing the number of nuclei that can grow to branches.

### Growth of NTs under high solution concentration (step 3c)

Now we consider the high solution concentration (*i.e.* zinc nitrate and HMT concentrations between 25 mM and 100 mM, for both reagents) part of step 3c, *without* the application of an electric field. We study growth for 30 min, since this relatively short time avoids the dominance of the growth of the parent rods over the branches. Branches with larger diameters form for the higher concentration solution *without* the electric field because high concentrations of reactants decrease the critical nuclei size as well as promote the growth rate.

Fig. 2b displays the NT structure produced by LT-HM using 100 mM concentration zinc nitrate with an equivalent amount of HMT aqueous solution. The other parameters are identical to those for the growth of the NC array of Fig. 1iii. The lengths and diameters of the branches on the head and side-walls are, on average, ∼400 nm and 80 nm, respectively, compared to ∼50 nm and ∼10 nm for the lower concentration solutions (Fig. 1iii).

### Analysis of PEC water splitting performance of NR *vs.* NC *vs.* NT structures

**Fig. 3:**
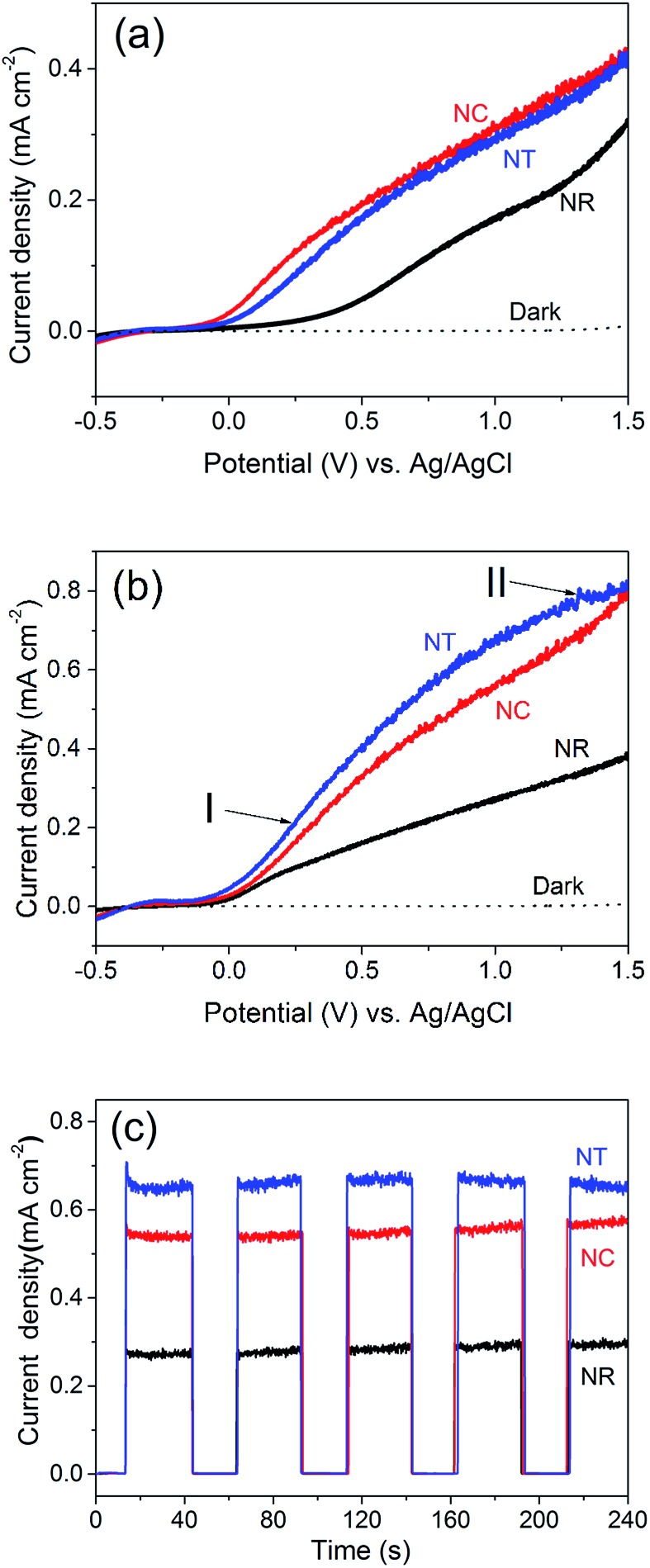
Linear sweep voltammetry (LSV) curves of the ZnO NR, NC and NT photoelectrodes (a) before and (b) after 450 °C annealing for 2 h in an ambient air atmosphere. I and II in (b) indicate the different parts of the LSV curve of the NT photoelectrode at low (I) and high (II) applied potentials. (c) Photocurrent density–time (*J*–*t*) curves of the ZnO photoelectrodes of (b). These were measured at an applied bias of 1.0 V *vs.* Ag/AgCl.

We now turn to the PEC water splitting performances of the different ZnO nanostructures, *i.e.* the NR (Fig. 1i), NC (Fig. 1iv) and NT (Fig. 2a) photoelectrodes. The measurements were investigated in a three-electrode PEC cell. The LSV performance was compared before (Fig. 3a) and after (Fig. 3b) annealing of the structures at 450 °C for 2 h in an ambient air atmosphere. We note that the photocurrent originating from the PEC water splitting can be directly correlated with the amount of hydrogen evolved.^48,49^

Before annealing (Fig. 3a), we see that the NC and NT structures both show improved performance over the NR structures. The photocurrent density of the NT photoelectrodes is 0.01–0.03 mA cm^–2^ lower than the NC photoelectrode even though the NT structure possesses more branches than the NCs. This may be due to the greater concentration of OH^–^ induced by the applied electric field during the NT growth process.

**Fig. 4:**
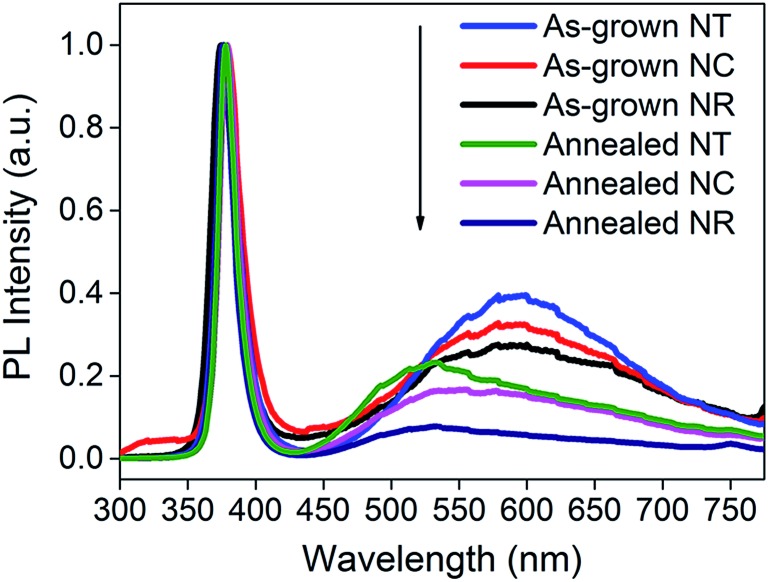
PL spectra of ZnO NRs, NCs and NTs before and after 450 °C annealing. The spectra are normalized to the peak values of the UV emission bands.

After annealing (Fig. 3b), all the structures give improved performance. This is attributed to a decreased defect concentration which causes carrier recombination.^50^ Fig. 4 shows the PL spectra for all the ZnO nanostructures (before and after annealing). The spectra consist of a UV peak centered at 378 nm which can be attributed to exciton recombination^51,52^ and a broad visible emission band which can be related to oxygen vacancy (V_o_) defects.^53–56^ The decrease of the intensity of the visible emission band after annealing indicates the effective reduction in concentration of the V_o_ defects. The intensities of the visible emission band for the NCs and the NTs were higher than those for the NRs before and after 450 °C annealing, consistent with the presence of more defects at the interfaces between the branches and the parent rods. The photocurrent of the ZnO NC and NT structures is 0.56 and 0.67 mA cm^–2^ at an applied potential of 1 V *vs.* Ag/AgCl, respectively, which is more than double that of the NR structures (0.28 mA cm^–2^) and previously reported pristine ZnO NR structures prepared by a similar deposition method (∼0.25–0.3 mA cm^–2^).^38,39,41^ Although the photocurrent of the ZnO NT structure is lower compared to the highest photocurrent of a similar ZnO NT reported by Xing Sun *et al.* (*i.e.* 0.67 mA cm^–2^ *cf.* ∼0.82 mA cm^–2^, both at 1 V), the much shorter fabrication period (less than 1 h *vs.* 17 h) of our NT structures gives much greater potential for large-scale application.^44^

Amperometric *J*–*t* studies were performed to examine the photoresponse of the NR, NC and NT photoelectrodes over time. *J*–*t* curves with light on/off cycles at 100 mW cm^–2^ at 1 V are shown in Fig. 3c. The data show very low dark current densities lower than 10^–3^ mA cm^–2^ for all three photoelectrodes. Upon illumination with light, the NR and NC photoelectrodes do not reveal a decaying trend. Only the photocurrent density of the NT electrode shows a bit of decrease (∼10^–2^ mA cm^–2^ in 240 s). This indicates that the ZnO nanostructures are relatively stable in the PEC water splitting process in a mild aqueous solution (0.5 M Na_2_SO_4_, pH = 5.8) at an applied bias of 1 V *vs.* Ag/AgCl. In the long term, coating of a thin protective shell such as TiO_2_ could guarantee the chemical stability of ZnO in more basic solutions to further improve the PEC water splitting performance.

It is worth noting that the rate of the increase of photocurrent with voltage for the annealed NT photoelectrodes is lower compared to that for the NC photoelectrodes (Fig. 3b). For the NC photoelectrode, the part of the LSV curve ranging from 0 to 1.2 V is fit well by the Gärtner model:^57^ *i*_ph_ ∝ exp(–*κW*) ∝ exp[*κ*(*E* – *E*_FB_)^0.5^]where *i*_ph_ is the photocurrent, *κ* the optical absorption coefficient, *W* the thickness of the space charge layer, and *E*_FB_ the flat-band potential. For the NT photoelectrode, the curve cannot be fit well to the model.

**Fig. 5:**
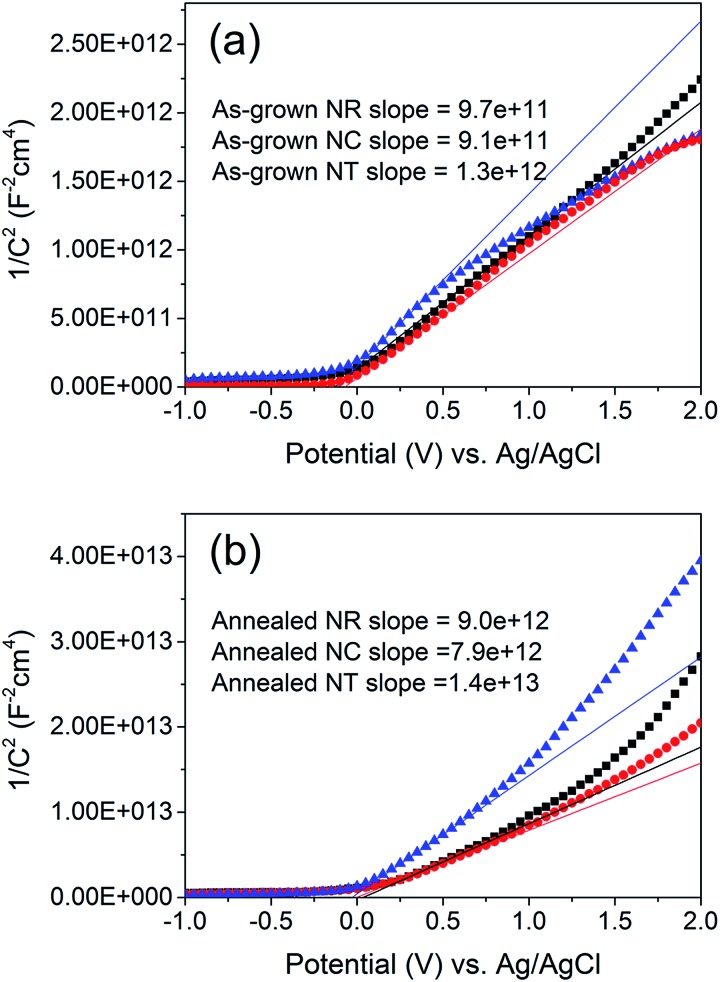
Mott–Schottky plots of (a) as-grown and (b) 450 °C annealed ZnO NR, NC and NT (square, circle and triangle, respectively) photoelectrodes. The NR, NC and NT arrays were deposited using identical parameters to those of the samples shown in Fig. 1i, iv and 2a. The estimated surface area of the NR, NC and NT arrays is approximately 3, 6 and 20 cm^2^ for a 1 cm^2^ working electrode, respectively. The solid lines represent the extrapolated lines from the linear portion of the Mott–Schottky plots.

In order to understand the underlying causes for the difference between the LSV curves of the annealed NC and NT photoelectrodes, the intrinsic electronic properties of the ZnO nanostructures, including the carrier density *N*_D_ and the flatband potential *E*_FB_ (Table S1†) at the nanostructure/electrolyte interface, were determined by measuring the space-charge capacitance per unit area of interface *vs.* the electrode potential (Fig. 5). The relationship between capacitance *C* and electrode potential *E* varies according to the Mott–Schottky equation:^58^1/*C* ^2^ = (2/*qεε*_0_ *N*_D_)[(*E* – *E*_FB_) – *kT*/*q*]where *ε* = 10 is the dielectric constant of the ZnO layers, *ε*_0_ = 8.85 × 10^–14^ F cm^–1^ the vacuum permittivity, *q* = 1.6 × 10^–19^ C the positive elementary charge. The carrier density can be estimated with the slope determined from the analysis of Mott–Schottky plots using the equation*N*_D_ = (2/*qεε*_0_)[d(1/*C* ^2^)/d*E*]^–1^

The thickness of the space-charge layer (Table S1†) can be derived from the Mott–Schottky plot relationship and is described by the equation^36^ *W* = [2*εε*_0_(*E* – *E*_FB_)/*qN*_D_]^1/2^

Using the *N*_D_ values (Table S1†) resulting from the Mott–Schottky measurements, the space charge thickness *W* at *E* – *E*_FB_ = 1 V was obtained as approximately 6 nm for the annealed NC photoelectrode and 9 nm for the annealed NT one. Since the value of 9 nm is larger than the radius of the branches in the NT structure, it can be concluded that the photocurrent is limited naturally by the geometrical limit of the radius of the branches of the NT structures (Fig. S5†).

**Fig. 6:**
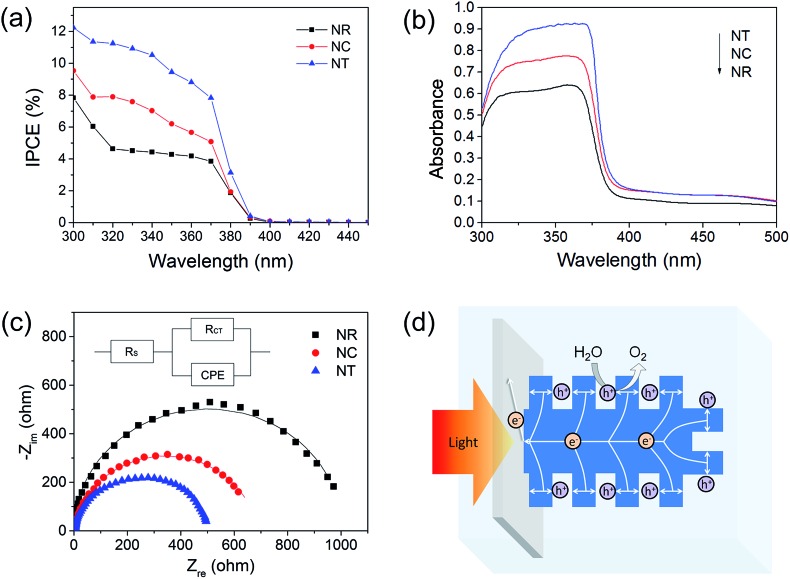
(a) IPCE spectroscopy at 0 V *vs.* Ag/AgCl; (b) UV-vis absorbance spectroscopy of the 450 °C annealed NR, NC and NT photoelectrodes; (c) Nyquist plots of the photoelectrodes under illumination. The solid lines represent the fitted curves of the measured data to the equivalent circuit model; the inset shows the equivalent circuit; (d) schematic illustration of the charge transport in the ZnO NT photoanode under illumination.

To provide insight into the PEC material properties, IPCE, UV-vis absorbance and EIS measurements were carried out. The IPCE results (Fig. 6a) for all the photoelectrodes annealed at 450 °C are NTs > NCs > NRs, which are consistent with their corresponding PEC performances shown in Fig. 3b. The results also indicate that the enhanced photocurrent mainly results from the UV light response. It is known that IPCE is affected by the efficiencies of three fundamental processes involved in PEC:^59^IPCE(*λ*) = [*η*_e^–^/h^+^_(*λ*)][*η*_collection_(*λ*)][*η*_transport_(*λ*)]where *η*_e^–^/h^+^_ is the efficiency of charge generation, *η*_collection_ the efficiency of charge collection (transfer) at the electrode/electrolyte interface, and *η*_transport_ the efficiency of charge transport within the material.

It is known that the charge generation efficiency is closely related to the amount of light absorption.^37^ The UV-visible absorbance spectra (Fig. 6b) show that the absorptions ranging from 300 to 380 nm increase for the NC and NT structures compared with the NR structure. The estimated surface area of the NC and NT arrays is approximately 6 and 20 cm^2^ for a 1 cm^2^ working electrode, respectively, an increase of 1 and 5.67 times when compared to the NR array. Thus, the increase of light absorption can be attributed to the increased volume filling and the increased multiple refection effect of the branches.^38^

The charge transfer process of the photoelectrodes was studied by EIS under illumination. Fig. 6c presents the Nyquist plots of the PEC system using the ZnO NR, NC and NT photoelectrodes after 450 °C annealing treatment under standardized solar-light illumination. The Nyquist plots were fitted to the equivalent Randle circuit as shown in the inset in Fig. 6c, where *R*_S_, CPE and *R*_CT_ represent the electrolyte solution resistance, the constant phase element for the electrode/electrolyte interface, and the interfacial charge transfer resistance across the electrode/electrolyte, respectively.^60^ A lower value of *R*_CT_ indicates a more efficient charge transfer across the electrode/electrolyte interface, reducing the possibility of charge recombination.^61^ For the 450 °C annealed photoelectrodes, the fitted *R*_CT_ values were NTs < NCs < NRs, which correspond well with the IPCE results of the ZnO photoelectrodes shown in Fig. 6a, indicating that the larger surface-to-volume ratios of the hierarchical nanostructures were of benefit to the charge transfer process across the interface to the electrolyte. Compared with the NR structure, the NC and NT structures with larger surface-to-volume ratios can supply an increased area of interfacial reaction sites, and increased area of depletion region (Fig. S5†) for separating the generated electron–hole pairs, which improve the efficiency of charge collection (transfer) at the electrode/electrolyte interface.

In terms of the efficiency of charge transport within the material, both NCs and NTs are expected to be better than NRs due to their thin branches. The charge transport within the branched ZnO photoelectrode is illustrated in the case of the NT photoelectrode as shown in Fig. 6d. Under irradiation, photo-generated electrons and holes transport to the cathode through the external circuit and to the ZnO/electrolyte interface, respectively. Gaseous O_2_ molecules are created at the ZnO/electrolyte interface due to the oxidation of O^2–^ by the holes. Evidently, the transport of the holes to the ZnO/electrolyte interface in the ultrathin branches of the NTs is more effective than that in the NRs as most electron–hole pairs are formed within the diffusion length of the ZnO/electrolyte interface.

## Conclusions

In this work, we demonstrated a versatile route to fabricate hierarchical ZnO nanostructures which give rise to high photocurrents. Both ZnO nanorod cluster (NC) and nanotree (NT) arrays from the parent ZnO NR structures were formed. The ultimate nanostructure morphology depends on competitive growth between the ZnO parent NRs and branches which are grown by low temperature hydrothermal growth on ZnO seed-coated NRs. A NC structure formed when a partially covered seed layer was applied to the NRs. On the partially covered seed layer, high solution concentrations or application of an electric field gave rise to a NT structure. When a completely covered seed layer was applied to the NRs, a NT structure was always formed. An analysis of the nucleation and growth on the different NR surfaces under the different growth conditions was made to explain the formation of the different structures.

The PEC response of the nanostructures, after annealing at 450 °C to optimize their performance, was in the order NTs > NCs > NRs. Both the NC and NT photoelectrodes displayed superior PEC behavior (0.56 and 0.67 mA cm^–2^ at 1 V *vs.* Ag/AgCl) compared to the NR structures owing to their much larger surface-to-volume ratios. Moreover, the NT photoelectrode with prominent PEC behavior also possesses a much shorter fabrication period (∼1 h compared to >10 h in the literature). The superior PEC water splitting performances of the NT and NC photoelectrodes were shown to originate from enhanced UV light absorption owing to increased volume filling and increased multiple refection effect, improved charge transfer process across the interface to the electrolyte because of increased area of interfacial reaction sites and increased area of depletion region for separation of generated electron–hole pairs, and efficient charge transport within the material due to the thin arms of the branched nanostructures where most electron–hole pairs are formed within the diffusion length of the ZnO/electrolyte interface.
