# Supplementary material for: Photoelectrochemical water splitting strongly enhanced in fast-grown ZnO nanotree and nanocluster structures
Source: J Mater Chem A Mater. 2016 Jun 1;4(26):10203–11. doi: 10.1039/c6ta02788a (PMC5059783; doi:10.1039/c6ta02788a)
Supplement: Supplementary file 1 [file TA-004-C6TA02788A-s001.pdf]

Electronic Supplementary Material (ESI) for Journal of Materials Chemistry A.
This journal is © The Royal Society of Chemistry 2016

Supporting Information
Photoelectrochemical Water Splitting Strongly Enhanced in Fast-Grown ZnO Nanotree and Nanocluster Structures

Xin Ren*[a], Abhijeet Sangle[b], Siyuan Zhang[b], Shuai Yuan[a], Yin Zhao[a], Liyi Shi[a], Robert Hoye[b], Seungho Cho[b], Dongdong Li[c], Judith L. MacManus-Driscoll*[b]

[a]*Research Center for Nanoscience and Technology, Shanghai University, 99 Shangda Road, Shanghai 200444, China. *Email: renxin108@shu.edu.cn; Tel: +86-21-66137197; Fax: +86-21-66137197*

[b]*Department of Materials Science and Metallurgy, University of Cambridge, 27 Charles Babbage Road, Cambridge CB3 0FS, UK. *Email: jld35@cam.ac.uk; Tel: +44-1223334468; Fax: +44-1223334468*

[c]*Shanghai Advanced Research Institute, Chinese Academy of Sciences, 99 Haike Road, Zhangjiang Hi-Tech Park, Shanghai 201210, China*

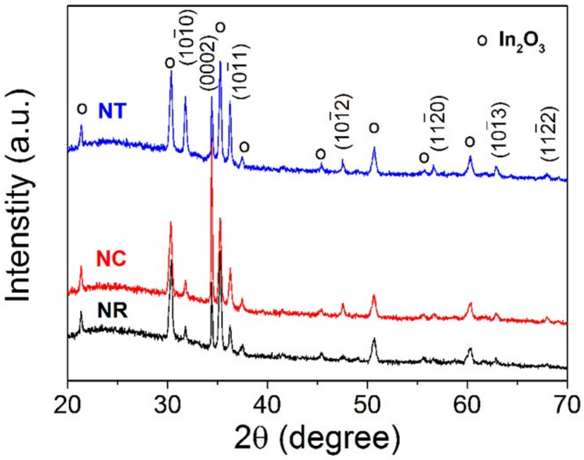

Fig. S1 XRD patterns of ZnO NR, NC and NT arrays deposited on ITO substrates corresponding to Figs. 1i, 1iv and 2a, respectively, indexed according to the hexagonal wurtzite structure (JCPDS No. 36-1451). The peaks associated with In_2O_3 from ITO substrates are labeled for clarity.

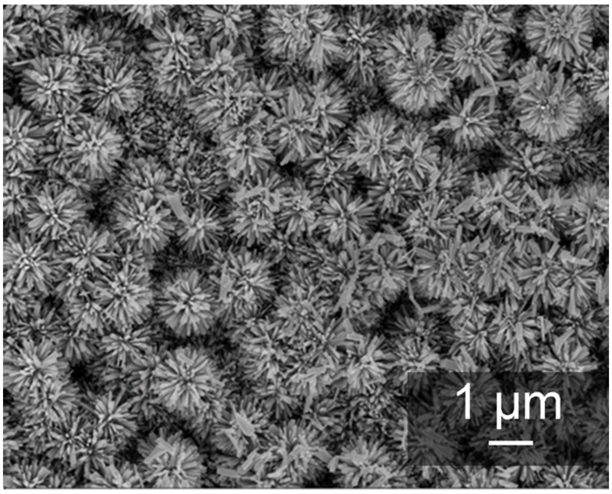

Fig. S2 Top view SEM image of ZnO NT arrays produced by the same fabrication approach as the NC array shown in Fig. 1iv, except step 2b was used instead of step 2a. In step 2b, the ZnO seed layer was deposited by atmospheric pressure chemical vapor deposition (AP-CVD). In short, diethyl zinc vapor was the Zn precursor and was mixed with nitrogen gas flowing at 150 mL min^{-1} through the metal precursor line; H_2O was the oxidant and was also mixed with nitrogen gas flowing at 150 mL min^{-1} through the oxidant line. Nitrogen gas was also directly fed to the AP-CVD manifold at 750 mL min^{-1} to provide the inert gas channels. The number of oscillations was 20, and the estimated thickness of the AP-CVD layer was 20 nm.

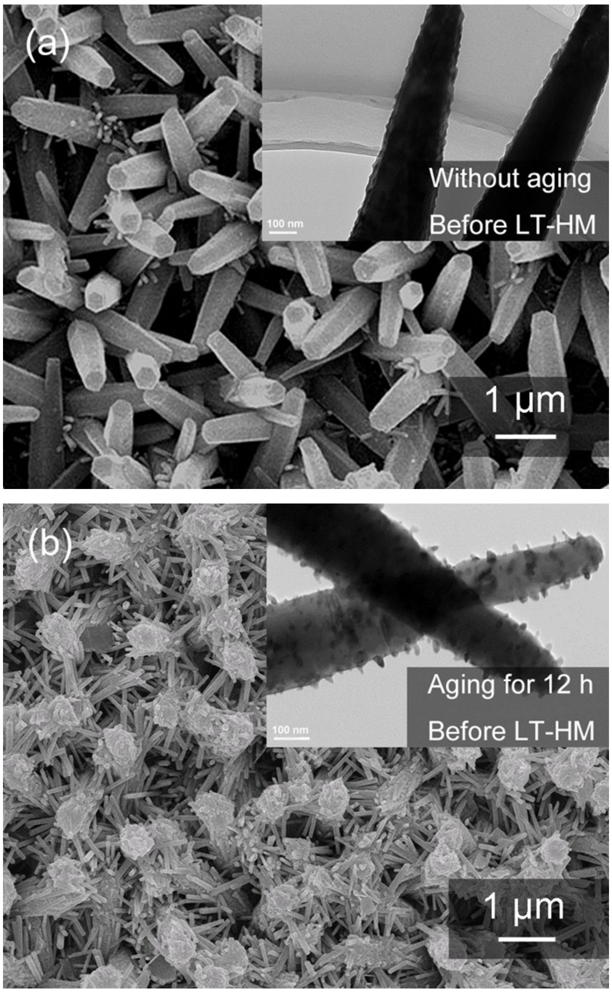

Fig. S3 Top view SEM images of ZnO NT arrays produced by the same fabrication approach as the NC array shown in Fig. 1iv, except step 2c was used instead of step 2a. In step 2c, the ZnO seed layers (the insets of Fig. S3a and S3b) were deposited by sol-gel. In short, the sol was 0.75 M zinc acetate dehydrate and an equivalent amount of monoethanolamine in a 2-methoxyethanol solution, mixed for 2 h at 60 ^{o}C. After dipping in the sol for 20 s, the seeded NR samples were taken out and divided to two series. One was put in to a muffle furnace immediately and annealed at 550 ^{o}C in air for 1h (the inset of Fig. S3a). The other was aged in dark at room temperature for 12 h, then was treated with the identical annealing process (the inset of Fig. S3b). Fig. S3a and S3b display the ZnO NT arrays grown from the different seeded ZnO NR shown in the insets of Fig. S3a and S3b, respectively.

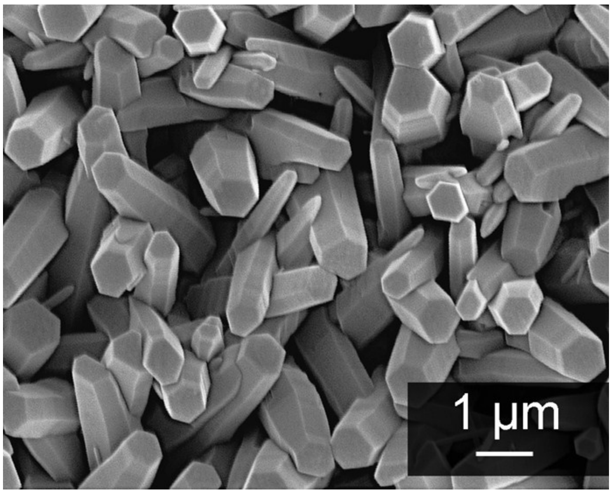

Fig. S4 Top view SEM image of ZnO NR array produced by the same step 1 and step 3a fabrication approach as the NC array shown in Fig. 1iv, except step 2 was cancelled.

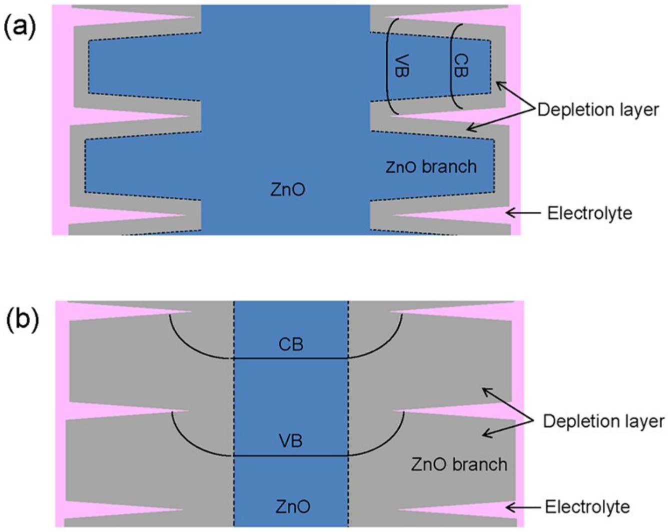

Fig. S5 Schematic representation of space charge region of the ZnO NT at the photoelectrode/ electrolyte interface for (a) low and (b) high applied potentials corresponding to Fig. 3b I and II.

Fig. S5 illustrates the space charge regions of the ZnO NT at the photoelectrode/ electrolyte interface for (a) low and (b) high electrode potentials. Fig. S5a shows that at low potential, the depletion layer generated in the ZnO only partly fills the branches from the interface between the NT and the electrolyte. With an increase of the applied potential, the depletion layer expands toward the inner core of the branches. Fig. S5b shows that at high potential, the depletion layer has filled the entire branch width, and begins to expand into the ZnO parent rods. It can be seen that the increased area of the space charge region at low electrode potential is much higher than that at high potential for the same increase of the space charge thickness (ΔW).

Table S1 Intrinsic electronic properties of the ZnO nanostructures derived from Fig. 5

Sample	N_D (cm^{-3})	E_{FB} (V) vs. Ag/AgCl	W (nm)
As-grown NR	2.3E+20	-0.13	2
As-grown NC	2.5E+20	-0.07	2
As-grown NT	1.8E+20	-0.12	3
Annealed NR	2.5E+19	0.04	7
Annealed NC	2.9E+19	0.00	6
Annealed NT	1.6E+19	-0.03	9
